# Flip a coin: Collaborative consumption or Sharing Economy in Ophthalmology services

**DOI:** 10.22336/rjo.2021.68

**Published:** 2021

**Authors:** Iuliana Raluca Gheorghe, Victor Lorin Purcărea, Consuela-Mădălina Gheorghe

**Affiliations:** * Department of Marketing and Medical Technology, “Carol Davila” University of Medicine and Pharmacy, Bucharest, Romania

**Keywords:** sharing economy, collaborative consumption, ophthalmology services

## Abstract

The sharing economy and collaborative consumption provide new opportunities in health care, especially to physicians, medical organizations, and consumers (patients). In health care services, there has been a long debate on the implementation and the characteristics of such concepts. Both terms may be very cost-effective, if applied accordingly.

The aim of this paper was to investigate whether the concepts of sharing economy and collaborative consumption can be successfully used in health care services, with a specific interest in Ophthalmology services.

The conclusions reached revealed that sharing economy in Ophthalmology services should be used from a consumer perspective, whereas the collaborative consumption concept should be used from a provider’s perspective.

## Introduction

Today, the upsurge of digital technology in health care services and other fields has visible outcomes, in the growing usage of digital platforms and platform providers [**1**]. 

As consequence, the concepts of Sharing economy (SE) and Collaborative consumption (CC) emerged and have become more useful in the Web 2.0 context. The rising popularity of both sharing economy and collaborative consumption has attracted significant research debates [**2**], and, recently, experts have identified how these emergent forms of consumption may challenge the key elements of Marketing, namely organizations, processes or interactions and value creation, emphasizing the need to address the topic in an in-depth approach. So far, scientific literature revealed that these forms of consumption are based on peer-to-peer interaction [**3**], and highlighted the antecedents of consumers engagement in these exchanges [**4**]. 

The determinants of collaboration between individuals and the predisposition to sharing can be evaluated according to the theoretical assumptions of collective action. Pizzol et al. [**5**] argue that individuals must undergo external pressure to act in a partnership manner in building and managing common goals in order to secure their own long-term interest. Moreover, these concepts include five main elements, namely [**6**]: value creation, underutilized assets, online accessibility, community, and reduced need for ownership. 

Given that the concepts SE and CC are new, a misunderstanding of their meaning prevails both in literature and practice, among consumers and service providers, being used interchangeably [**7**]. While the two concepts present some common characteristics, such as the adoption of temporary access for using goods and services, and the dependence on the Web 2.0 access [**8**], they also present differences. For instance, sharing has been an exchange method based on trust, bond, generosity, and reciprocity, whereas, collaborative consumption presents the existence of a triadic exchange that takes place between a platform provider, a peer service provider, and a consumer, with the aim of making profit or benefit from a form of compensation [**4**].

Research in this area has concentrated mainly on the hotel industry [**9**] and on car renting [**10**], but we believe that the peer-to-peer collaborative consumption or sharing consumption is very important in health care services as well [**11**]. Although healthcare services have many particularities that make them challenging for specialists, sharing or collaborating would surely lead to satisfied consumers and medical staff. To enumerate a few benefits of using collaborative consumption or sharing economy, health care organizations may ease the management of the doctor-patient relationship by creating websites on which health care consumers can find, compare, and book practitioners directly, and, at the same time, practitioners will be rated according to the reviews of the patients. Moreover, health care services require practical collaborative support from multiple parties to fulfill the real-time information necessities of daily clinical care and provide prompt accessibility to patient care. Since there are not many medical platforms for sharing economy or collaborative consumption, this could be an underexplored and potentially very profitable future market for the medical field. 

The aim of this paper was to investigate whether the concepts of sharing economy and collaborative consumption can be successfully used in health care services, with a specific interest in Ophthalmology services. 

## Collaborative consumption or sharing economy in Ophthalmology services

The pandemic context and the lack of medical staff in all health care fields and medical devices that are very expensive to own led to a need for reduction of medical expenses in the health care system [**12**]. 

Sharing in the health care field has a lot of history behind and has been researched under different names in the Marketing literature, as for example user-generated content or electronic word-of-mouth [**13**]. Sharing can be distinguished from other methods of consumption, especially in the absence of profit-oriented aims, as well as by the presence of the sense of community, generosity, and reciprocity [**4**]. 

As sharing has always depended on some form of network [**7**], and in health care, on communities, the recent upsurge of digital platforms that resulted from the digital usage proliferation significantly contributed to new ways of sharing and redefined the old ones [**8**]. Moreover, in health care services, digital or online communities evolved into Patient Online Communities (POCs) and, consequently, in patient support networks. 

The key to have a sustainable sharing activity in health care services is to keep health care consumers engaged with the platform and to deliver value at an information level, because, in practice, patients become co-producers in delivering a medical service [**14**]. As such, sharing economy is based on the creation value within the ecosystem [**15**]. 

For example, health providers, patient online communities (POCs), different advocacy organizations and patients’ peers (family, employers) may facilitate and encourage other patients to value co-creation activities by understanding procedures, treatments and symptoms and engage, in their turn, in other preventive and support activities for newly ill patients. Specifically, value creation has also been investigated in the context of Ophthalmology services [**16**]. However, today we are facing an evolved form of value creation, that is consumer value co-creation [**17**]. 

Value co-creation is described as a “collaborative social activity involving the exchange and integration of valuable resources and emphasizes the value determination of agents in particular domains or contexts [**18**]. More exactly, value is perceived by consumers based on the “value in use” rather than the “value in exchange”, as determined by providers, and so, the consumer will always be involved in the creation and the co-creation of value [**19**]. We find it appropriate to include here the conceptual model for the structure of consumer value in services, as presented by Sanchez-Fernandez et al. [**20**]. Their framework includes economic, social, hedonic, and altruistic value categories. The most relevant in the context of health care services are the economic and altruistic values. The economic value comprises goals of producing or co-producing efficient and excellent services, whereas, the altruistic value refers to “the other oriented consumption experience valued intrinsically for its own sake or as an end in itself” [**20**].

In Ophthalmology services, there are a few dedicated patient online communities supported by web platforms or mobile apps that have transformed into peer-to-peer networks. Some of them focus on fostering a peer coaching culture that offers consumers the chance to share stories and experiences about treatment, symptoms and regimes, and providers to engage in supporting or sharing constructive information. Apart from getting informed, educated and prevent certain ophthalmologic diseases, these forms of interaction generate real-time networks that could evolve in research databases. Moreover, there are also peer-to-peer networks dedicated to specialists in Ophthalmology, as for example, the Social Media closed groups that allow them to receive feedback of the complex cases from their clinical activity by posting relevant images and information.

Another example of a sharing economy is the home care provided by a personal support network of family, friends, and medical professionals. In Ophthalmology, this is encountered in the case of older people suffering from glaucoma. This network is built around the idea that the family and friends of an individual would want to help, based on the assumption of reciprocity, namely, “I will help you, so that someone will help me when I need assistance”. Since there is little room for family and specialists to meet face to face, the support offered on an online platform would be very efficient in this case. However, this should work provided that the medical professional is the physician of an individual and knows his medical history.

Collaborative support is necessary within health care organizations such as hospitals, because patients’ records must be moved among healthcare professionals. Moreover, collaboration among health care organizations is also very important for the patients who are being transferred from one health care provider to another for specialized treatments. This type of collaboration within or among health care organizations has been used to provide cost-effective outcomes [**21**], by using collaborative technologies [**22**]. 

A very famous collaborative platform dedicated to health care is Cohealo® [**23**]. It provides a technology-mediated sharing platform that allows infrastructure usage facilities to health care organizations without owning them, as follows:

• Cohealo + C-suite that allows the management of equipment related data. 

• Cohealo + Finance offers the possibility to optimize the utilization of their most important technology investments. 

• Cohealo + clinical is network-based application that allows health care organizations to locate any piece of medical equipment and enlist it in supporting a procedure, whenever it is needed. 

• Cohealo + supply chain facilitates the equipment delivery logistics by sending it where it is needed in optimized time and after usage return it to the initial location.

Another example of collaborative platforms is related to telemedicine, namely, the telehealth platforms, that present benefits to both consumers and physicians. In Ophthalmology, there are a lot of platforms dedicated to telemedicine, which have been used by health care consumers or patients, especially in the pandemic context. Thus, patients may seek telemedicine ophthalmologic services for episodic care, as for instance, in the case of a dry eye disorder, or may address more specific requirements necessary in the management of an eye disease, such as cataract. By facilitating information collection and offering it in a transparent manner, telehealth platforms have the potential to empower consumers, as well as protect their personal data. These online interactions may prove to be convenient concerning time and accessibility, but troublesome for the consultation procedure, the remote online examinations, message exchanges, video chats. In contrast, physicians can also benefit from telemedicine or the online collaborative platform in terms of work-life balance activities, due to flexible practice hours and locations. Today’s telehealth platforms have developed so much and are so innovative that they regulate quality and safety standards, screen and select providers, and give the consumers the possibility to rate the providers from many perspectives.

Further ideas of future directions of collaborative activities in Ophthalmology relate to providing access to very expensive medical equipment to health care organizations without owning them and adjusting a platform to keep track of them. The possibility to enlist machines and medical equipment, along with a contact person and an available location, should be found on the platform. Users will search for a particular medical equipment and the web platform will provide the most appropriate location. 

## Conclusions

With the rise of health technologies, it is appropriate to change the way we think about the traditional health care access because new models of economies have emerged and are still developing. In the light of getting more digitalized and since many health care activities moved in the online environment, the sharing and collaborative activities appeared. In health care services, both sharing economies and collaborative consumption concepts create an ecosystem of economies. The ecosystem is based on the physician-patient sharing networks, alliances and sharing resources, such as medical equipment. Since health care is a highly specialized field of work and science, it calls for more specific and focused problem-solving issues. We concluded that in Ophthalmology the sharing economy concept is specific for consumers or patients, whereas the collaborative consumption is based on the partnership between and within health care organizations, by using web platforms in both cases, the former in a free and altruistic manner, and the later, requesting a financial compensation.
